# Modeling Pollinator Community Response to Contrasting Bioenergy Scenarios

**DOI:** 10.1371/journal.pone.0110676

**Published:** 2014-11-03

**Authors:** Ashley B. Bennett, Timothy D. Meehan, Claudio Gratton, Rufus Isaacs

**Affiliations:** 1 Department of Entomology and Great Lakes Bioenergy Research Center, Michigan State University, East Lansing, Michigan, United States of America; 2 Department of Entomology and Great Lakes Bioenergy Research Center, University of Wisconsin - Madison, Madison, Wisconsin, United States of America; Central China Normal University, China

## Abstract

In the United States, policy initiatives aimed at increasing sources of renewable energy are advancing bioenergy production, especially in the Midwest region, where agricultural landscapes dominate. While policy directives are focused on renewable fuel production, biodiversity and ecosystem services will be impacted by the land-use changes required to meet production targets. Using data from field observations, we developed empirical models for predicting abundance, diversity, and community composition of flower-visiting bees based on land cover. We used these models to explore how bees might respond under two contrasting bioenergy scenarios: annual bioenergy crop production and perennial grassland bioenergy production. In the two scenarios, 600,000 ha of marginal annual crop land or marginal grassland were converted to perennial grassland or annual row crop bioenergy production, respectively. Model projections indicate that expansion of annual bioenergy crop production at this scale will reduce bee abundance by 0 to 71%, and bee diversity by 0 to 28%, depending on location. In contrast, converting annual crops on marginal soil to perennial grasslands could increase bee abundance from 0 to 600% and increase bee diversity between 0 and 53%. Our analysis of bee community composition suggested a similar pattern, with bee communities becoming less diverse under annual bioenergy crop production, whereas bee composition transitioned towards a more diverse community dominated by wild bees under perennial bioenergy crop production. Models, like those employed here, suggest that bioenergy policies have important consequences for pollinator conservation.

## Introduction

Demand for sustainable sources of energy has spurred increasing interest in bioenergy crops as a fuel source. In the United States, government mandates to increase biofuel production to 36 billion gallons per year by 2022 are advancing research into the production and sustainability of both first- and second-generation biofuels [1], [2]. First-generation biofuels are produced from annual row crops such as corn, soybean, and canola, while second-generation cellulosic biofuels can be produced from corn stover, switchgrass, or mixed grasslands, a combination of warm-season grasses and forbs [3]. These contrasting options for biofuel cropping systems have the potential to dramatically alter the types and perenniality of vegetative cover in agricultural landscapes, significantly affecting wildlife [4]. Because policies that promote biofuel production have the potential to cause large scale changes in land use [5], [6], identifying how bioenergy crops affect biodiversity will be critical to developing sustainable biofuel policies appropriate for regional implementation across the United States.

A growing body of research suggests that bioenergy crops differentially support biodiversity. For example, bird abundance and richness was consistently higher in perennial grassland biofuel plantings compared to annual biofuel plantings, with expanded grasslands predicted to increase bird richness by 12–207% [4], [7]. Similarly, predatory arthropods increased in abundance and diversity in perennial grassland biofuel crops compared to corn monocultures [8], [9]. Transitioning the landscape into either annual or perennial biofuel crops will affect species diversity and community composition but also the provisioning of valuable ecosystem services, such as arthropod-mediated predation of crop pests [10], [11].

Wild bees, which provide $3.1 billion in pollination services annually to agricultural landscapes in the United States [12], are also expected to be affected by the type of bioenergy crops selected. Research at the field level has found bee abundance to be three to four times higher within perennial grasslands than in corn fields [8], while at the landscape level, pollinators respond positively to increasing amounts of natural area and negatively to landscapes dominated by annual agriculture [13], [14], [15]. The response of pollinators to land-cover change suggests that the selection of bioenergy crops for large-scale production has the potential to positively or negatively impact these organisms. Expanding production of annual bioenergy crops such as corn or soybeans would further simplify the landscape by increasing the proportion of monoculture plantings across landscapes, reducing the availability of food and nesting resources for pollinators. In contrast, expansion of perennial grassland bioenergy crops could benefit pollinators by increasing landscape heterogeneity and augmenting the amount of resource rich-habitat available for foraging and nesting by bees.

With interest in identifying viable bioenergy crops, predictive models have been employed to investigate the effect of different bioenergy crops on species of conservation concern such as grassland birds [4], as well as a range of ecosystem services including biocontrol, carbon sequestration, and phosphorous loading [11], [16], [17]. Because pollinators provide a valuable ecosystem service and many are experiencing declining populations [18], [19], models have also been developed to explore the effects of landscape composition on bee populations [20], [21], [22]. The conversion of land into more intensive uses is expected to have significant effects on pollinators [23], yet the use of modeling to predict the effects of intensive large scale land use change on pollinators is limited [24]. Because pollination is a critical service provided to agricultural crops and to natural plant communities [25], [26], bioenergy policies should proactively address how bees can benefit from the development of agricultural systems that advance crop production as well as conservation objectives.

In this research, our aim was to explore the potential effects of two different bioenergy crop production scenarios on pollinator communities using a modeling approach. First, using observations from flower-visiting bees across the state of Michigan, U.S.A, we developed models that related bee abundance, diversity, and community composition to the land cover surrounding study sites. The three community metrics we modeled can indirectly provide insights into how pollination services may be affected by changes in bioenergy production. For example, pollination services tend to increase as flower-visiting bees become more abundant and diverse [27], [28], [29]. Also, shifts in community composition that result from changes in bioenergy production may affect particular species known to be important pollinators or of conservation concern. Next, we used empirical models to predict the effect of different bioenergy production scenarios on bees. The bioenergy production scenarios tested in this study represented opposite extremes of possible future production scenarios, and assumed a transition to annual bioenergy crops or to perennial grassland bioenergy crops on marginal lands. Production scenarios were limited to marginal lands because a sustainable biofuel policy will likely need to minimize competition between lands devoted to food and biofuel production [30]. Because perennial grassland biofuel production would increase landscape diversity and incorporate more resource-rich habitats into the landscape [21], we predicted that bee abundance, diversity, and community composition would benefit from a biofuel policy that increases perennial grassland production.

## Methods

### Study Sites

**Figure 1:**
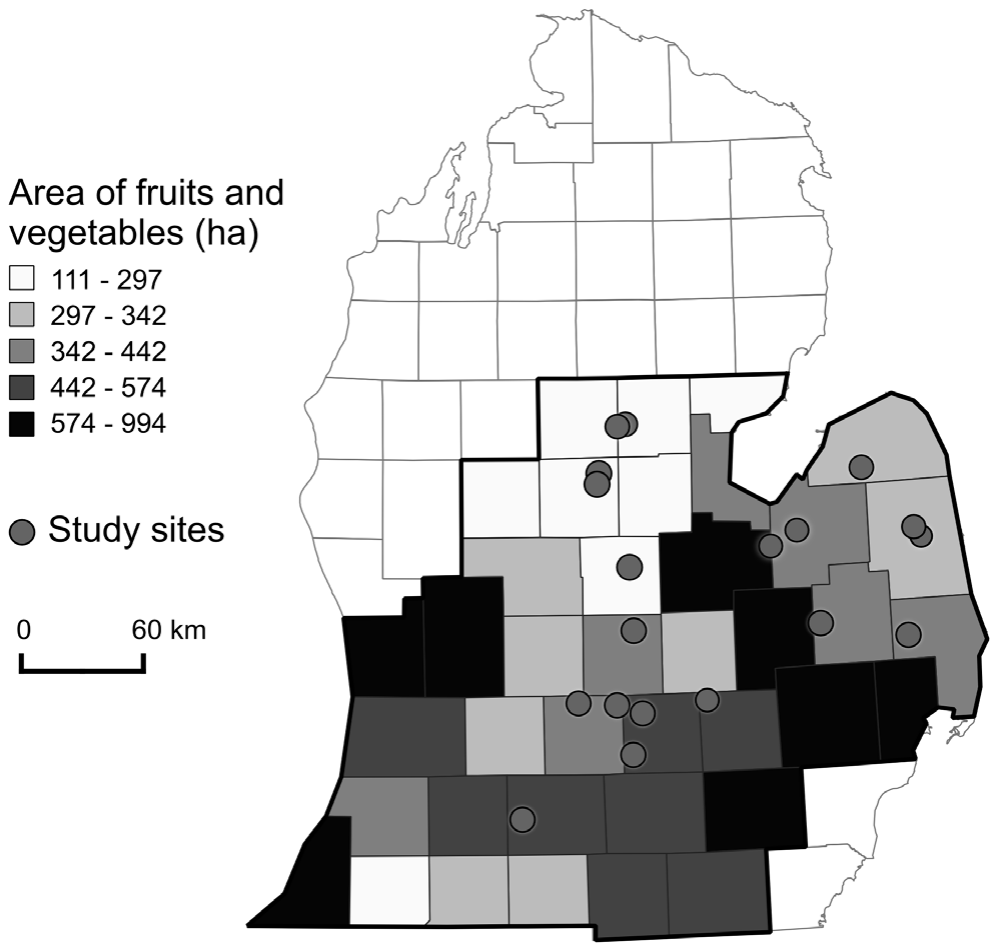
Study sites. Location of study sites sampled for bees across Michigan. Hectares of fruits and vegetables are calculated on a county basis and shown for the lower region of Michigan.

Field sampling was conducted with the permission of land owners in 20 soybean fields located across southern Michigan (Fig. 1) during the summer of 2012. We observed bee visitation at sentinel insect-pollinated plants to estimate bee abundance, diversity, and community composition. Sites were at least 3 km apart and varied in the proportion of annual crops and semi-natural habitats in a 1500 m radius surrounding each site. Land use proportions surrounding study sites were calculated using a geographic information system (GIS, ArcGIS, version 10.0, ESRI, Redlands, CA). The proportion of grassland in the landscape ranged from <1% to 60%, representing a range of possible biofuel production scenarios from sites dominated by annual production to those dominated by perennial grassland production. Bee observations were conducted in soybean fields across a landscape gradient because soybean is a flowering, first-generation biofuel crop that is intensively managed for even plant density and low plant diversity using weed control. This approach reduced variability in bee counts due to variation in flower abundance, plant diversity, and other management practices. However, this approach also requires that inferences about patterns in other crops be viewed with an appropriate degree of caution.

### Bee abundance, diversity and community composition

Bee visitation to sentinel sunflowers, *Helianthus annuus*, variety “Sunspot”, was measured at each study site to sample the pollinating bee community. Plants were grown in 15.2 cm diameter pots in a greenhouse under 24-h light and a temperature of 26.7±2°C. Two sunflowers with open disk flowers were placed at each of two sampling stations located 30 m from field edges and 20 m apart. Bee sampling was conducted simultaneously at each station during a 30-min period with one observer per station. Bees were collected using a hand-held vacuum (Bioquip, Rancho Dominquez, CA) when bee contact was made with disk flowers (i.e., anthers and/or stigmas). Each field (n = 20) was surveyed 3–5 times during the 2012 field season. Unequal sampling across sites was the result of agronomic activities which prevented access to fields on some dates. Sampling occurred on sunny days with temperatures above 24°C, and each field was sampled at least once in the morning and once in the afternoon. Bees were returned to the lab and identified to species using the online key to Bees of Eastern North America at www.discoverlife.org and published species-level keys [31], [32].

Bee abundance, diversity, and community composition were quantified for each site. Bee abundance was calculated by averaging the number of bees collected during a 60 min observation period (2 plants/site ×30 min) across all sampling dates for each site, and these values were square-root transformed prior to analysis to improve normality. To avoid obscuring the response of wild bees to landscape change, we excluded *Apis mellifera*, a managed pollinator commonly brought to agricultural landscapes, from calculations of abundance. Because *A. mellifera* was prevalent in agricultural landscapes and influenced composition (see Results), this species was included in calculations of bee diversity and community composition. Bee diversity was quantified for each site using Simpson's diversity index because this index is less sensitive to the degree of sampling effort (Simpson's diversity  = 1-D)[33] than species richness and other diversity indices such as Shannon's diversity.

The response of the bee community was then analyzed across sites. First, the abundance of each bee species at each site was averaged across the 2012 season and then square-root transformed and standardized by species and site (i.e., Wisconsin double standardization) [34]. The similarity between sites was then quantified using the Bray-Curtis coefficient. Bee community composition was then ordinated using non-metric multidimensional scaling (NMDS). Next, we explored the relationship between measured landscape variables (e.g., proportions of grassland, forest, wind-pollinated crops, and annual flowering crops; see below) and bee community composition using environmental vector fitting [34]. When viewing the plotted NMDS scores from the community ordination, along with the corresponding environmental vectors (i.e., landscape proportions), we found that most of the variation in bee communities, and most of the association between bee communities and landscape structure, was represented along the first NMDS axis. In order to draw a qualitative link between NMDS scores, bee community composition, and landscape composition, we identified the bee species that contributed most to differences in NMDS scores using a SIMPER (similarity percentages) analysis [34]. Here, the bee community at each site was classified as having a high (>0) or low (<0) NMDS score. The output of the SIMPER analysis elucidated those species that were most highly associated with the two ends of the NMDS spectrum. Given the association between NMDS and landscape gradients, the SIMPER output allowed us to understand how particular bee species responded to landscape composition. Following these preliminary analyses, we used the first NMDS axis values to represent bee composition in subsequent linear regression modeling, described below. NMDS ordinations, environmental vector fitting, and SIMPER analyses were performed using the vegan package [35] written for R statistical computing software [36].

### Modeling bee abundance, diversity, and community composition

Multiple linear regression was used to model bee abundance, diversity, and community composition as a function of land cover. Using the 2012 Cropland Data Layer (USDA NASS 2012), the proportion of land cover was calculated in the 1,500 m surrounding sites for nine classes, which accounted for 0.87±0.19 (SD) of land cover: annual wind-pollinated crops, which combined 24 classes of annual crops but was dominated by corn (average proportion of corn  = 0.60 in this category, based on all 20 sites); annual flowering crops, which combined 17 classes of annual crops that benefit from pollinators but were dominated by soybean (average proportion of soybean  = 0.89); perennial flowering crops (all fruit crops); grasslands (included herbaceous grasslands, old fields, pastures, wildflowers, hayfields, alfalfa fields, and shrublands); forests (combined deciduous, coniferous, mixed forest, and wooded wetlands); wetlands (herbaceous wetlands); suburbs (areas of low development and open areas dominated by turf); cities (areas of moderate to high development); and other (included water, walnut and Christmas tree farms, and barren). Wind-pollinated crops, annual flowering crops, forests, and grasslands were included as explanatory variables because they accounted for a large proportion of the land cover and (with the exception of forest) are the cover classes that will change under contrasting bioenergy scenarios. Although the proportion of forest does not change under the different bioenergy scenarios, this variable was retained in the model because forests are ecologically important to pollinators, providing nesting habitat and floral resources early in the season [37], [38], [39].

An information theoretic approach to model selection began with the full model, which included the proportion of wind-pollinated crops, annual flowering crops, grassland, and forest. The full model and all possible subsets of the full model were analyzed using the multimodel inference package, MuMIn, in R [36], [40]. The overall best model and all competing models were identified and ranked using bias-corrected Akaike's Information Criterion (AIC_c_). Because multiple competing models explained bee abundance, diversity, and community composition, we used model-averaged coefficients from the model set to make predictions about changes in bee communities under the different bioenergy scenarios. Model-averaged coefficients were calculated as weighted averages using model coefficients and Akaike weights, where coefficients were set to zero when a variable was not included in a given model (i.e., a shrinkage coefficient) [41]. Spatial autocorrelation in model residuals was assessed with spline correlograms using the ncf package in R [42]. Statistically significant spatial autocorrelation was not detected in model residuals (Text S1).

### Projecting bee abundance, diversity, and community composition

Bee abundance, diversity, and community composition were first estimated across the lower peninsula of Michigan under the current landscape scenario. In GIS, proportional land cover maps were calculated separately for each cover type using a moving window approach. The moving window analysis, which was preformed across Lower Michigan, calculates for each pixel the proportion of cover (e.g., grassland, forest, etc.) in a neighborhood with a radius of 1500 m. To generate predicted values for bees under the current land cover scenario, model-averaged coefficients from the empirical models were multiplied by their respective proportional maps. For example, the model-averaged coefficient for grassland was multiplied by the proportion grassland map and then summed with the products of other terms to produce predicted values for each pixel. The equation used to calculate predicted values for bees in GIS was as follows:
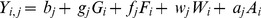
(eq.1)where *Y* is the prediction for the *i*th pixel for the *j*th bee community metric (i.e., bee abundance, diversity, or composition), *b_j_* is the intercept for the *j*th metric, and *g_j_*, *f_j_*, *w_j_*, and *a_j_* are the metric-specific model-averaged parameter values for the proportions of grassland, *G*, forest, *F*, wind-pollinated crops, *W*, and flowering annual crops, *A*, in the landscape surrounding the *i*th pixel. The results of this analysis generated maps that estimated bee abundance, diversity, and community composition under current landscape conditions.

The next step was to model abundance, diversity, and community composition under the two contrasting bioenergy scenarios. Because a sustainable bioenergy policy will need to minimize competition with highly productive agricultural land [30], the bioenergy scenarios we developed were focused on marginal land. The U.S. Department of Agriculture's SSURGO database, which lists land capability classes based on soil quality, erosion potential, and water saturation, was used to identify marginal land. Marginal land was defined as cropland with “severe limitations” to “very severe limitations” (land capability classes 3 and 4, respectively) in addition to other lands consider unsuitable for row crop production (classes 5–8).

Of the marginal land in Lower Michigan, we identified 1,200,352 ha in grassland, 550,750 ha in annual wind-pollinated crops (76% corn), and 290,033 ha in flowering annual crops (86% soybean). To keep the number of hectares converted in each scenario consistent, 600,000 ha of marginal lands were converted in each scenario. In the “perennial grassland bioenergy scenario” approximately 360,000 ha of wind-pollinated crops on marginal land and approximately 240,000 ha of flowering annual crops on marginal land were randomly selected and converted into grassland in GIS. In the “annual bioenergy scenario”, 600,000 ha of grassland on marginal land was randomly selected and converted into wind-pollinated crops or flowering annual crops based on the proportion of corn (0.58) and soybean (0.41) in the current landscape (CDL 2012). Once the land-cover conversions were completed in GIS, resulting maps represented land cover change under the perennial grassland bioenergy scenario and the annual bioenergy scenario.

The final step was to predict bee abundance, diversity, and community composition under the annual and perennial bioenergy production scenarios. First, we created proportional land cover maps for each cover type using the annual and perennial bioenergy scenario maps. Then model-averaged coefficients from the empirical models were multiplied by their respective proportional land cover maps (Eq. 1). Finally, we calculated the percent change in bee abundance and diversity between the current landscape (*Y_i,j,c_*) and each bioenergy scenario (*Y_i,j,s_*) using the equation: percent change  =  ((*Y_i,j,s_* – *Y_i,j,c_*)/*Y_i,j,c_*) ×100. Because bee composition used NMDS axis scores, which include both positive and negative values, the difference between the bioenergy landscape and the current landscape was calculated using: difference  =  *Y_i,j,s_* – *Y_i,j,c_*.

We produced aggregate summaries of the percent change in bee communities for each scenario across the study region in two distinct ways. First, we calculated summary statistics for changes in bee community metrics across all grassland and cropland pixels in the study region. Second, we calculated summary statistics for changes in bee community metrics only for changed pixels and their immediate neighbors across the study region. Hereafter, we refer to results from the former calculations as landscape-level results, and from the latter calculations as local-level results.

## Results

### Empirical data

**Figure 2:**
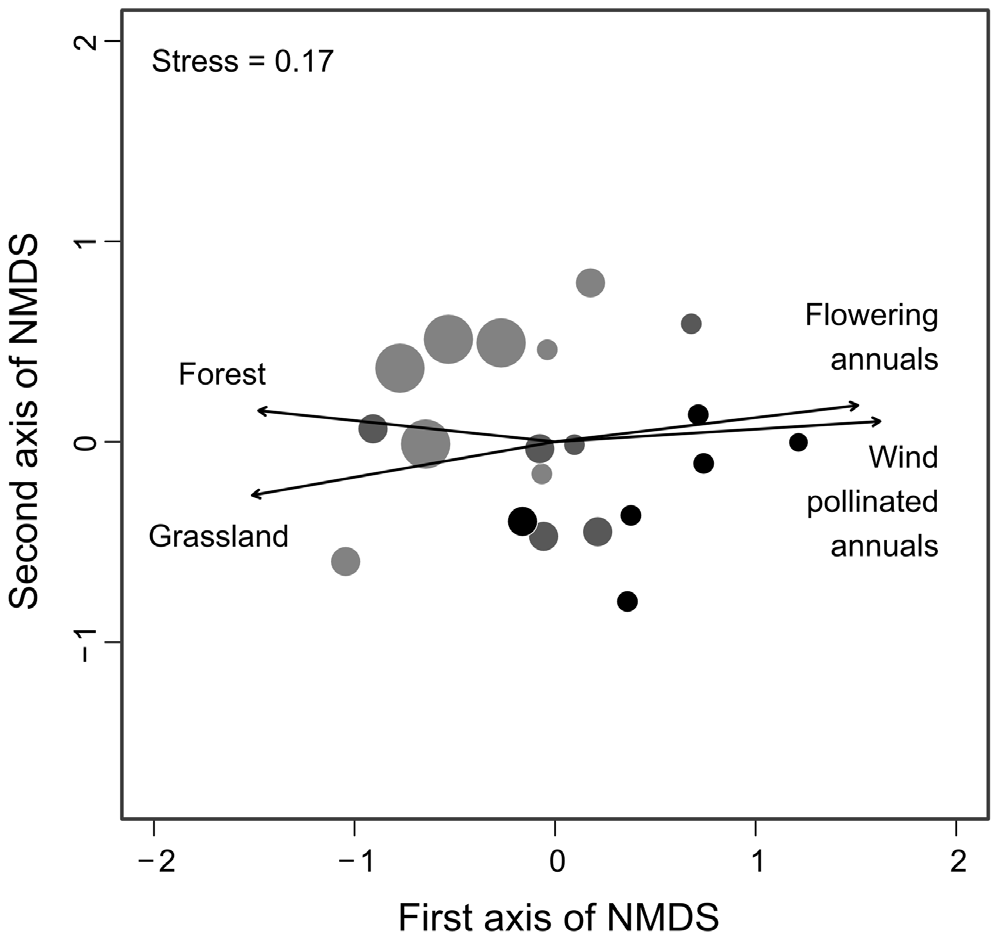
Ordination of bee communities with landscape variables. Ordination of bee community composition using non-metric multidimensional scaling (NMDS) shows that communities change as the proportion of grassland and forest cover increase in a 1500 m radius surrounding sites (stress  = 0.17). Sites with negative NMDS axis one scores are correlated with grassland (R^2^ = 0.46, *P* = 0.0054) and forest (R^2^ = 0.43, *P* = 0.0071), while sites with positive NMDS axis one scores are correlated with wind pollinated crops (R^2^ = 0.52, *P* = 0.0025) and flowering annual crops (R^2^ = 0.45, *P* = 0.0057). Increasing circle size represents sites with higher bee abundance while decreasing color intensity (black to light gray) indicates sites with higher levels of bee diversity.

During 4,800 min (80 hr) of observation on sentinel plants across all study sites, we observed an average of 0 to 7.25 bee visitors per hour. A total of 38 bee species were identified, and species richness ranged from 1 to 16 species per site, which translated into a range of Simpson's diversity values of 0 to 0.89 per site. The NMDS and vector fitting analyses showed that bee community composition changed along a landscape gradient (Fig. 2, two-dimensional stress  = 0.17), where communities in sites with low proportions of grassland and forest had positive NMDS axis scores, while communities associated with high proportions of grassland and forest had negative NMDS axis scores. The proportion of grassland and forest were significantly negatively correlated with the first NMDS axis (Fig. 2; Grassland: R^2^ = 0.46, *P* = 0.005; Forest: R^2^ = 0.43, *P* = 0.007), while the proportion of wind-pollinated and flowering annual crops were positively correlated with the first NMDS axis (Fig. 2; Wind: R^2^ = 0.51, P = 0.003, Flowering annual: R^2^ = 0.45, *P* = 0.006). In general, the bee communities in landscapes dominated by annual crops were less diverse while those in grassland and forest dominated landscapes were more diverse (Text S2). The SIMPER analysis indicated that *A. mellifera*, *Augochlorella aurata*, and *Halictus ligatus* contributed to the largest difference: 17%, 8%, and 7%, respectively, among sites along the landscape gradient (Text S2). *A. mellifera* and *A. aurata* contributed more to the bee community in agricultural landscapes while *H. ligatus* was increasingly associated with sites having higher proportions of grassland and forest in the surrounding landscape (Text S2).

### Modeling pollinators

**Figure 3:**
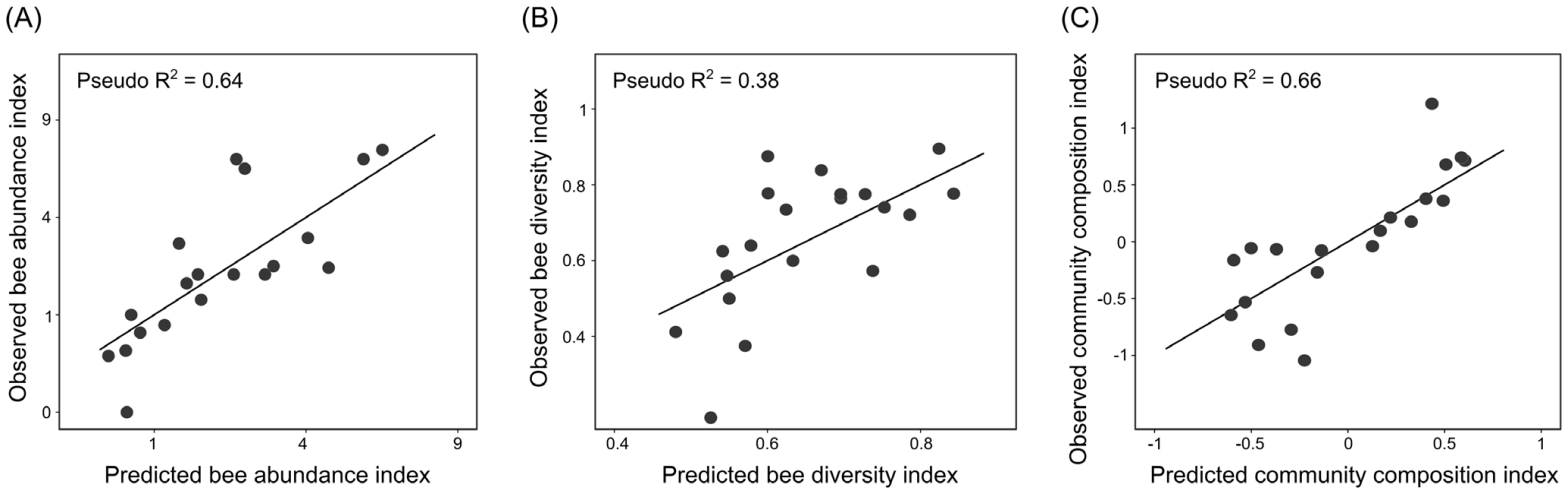
Observed versus predicted bee community metrics. Relationships between observed and predicted values for abundance, diversity, and community composition. Pseudo-R^2^ is derived from regressing observed values versus model-averaged predictions. The graph for abundance displays only 19 points because two data points overlap.

Bee abundance was best explained by the proportion of forest and grassland in the surrounding landscape (Table 1). Because several competing models were present, model-averaged coefficients calculated with shrinkage were used to predict and map bee abundance in GIS (Table 1, Fig 3A). Overall, forest and grassland had positive model-average coefficients for bee abundance, while wind-pollinated crops and flowering annual crops had negative model-average coefficients.

**Table 1:** Model selection.

	Intercept	Grassland	Forest	Wind^a^	FA^b^	ΔAICc	Model weight	Model R^2^
Abundance								
Competing model								
1	0.66	1.83	2.98			0.00	0.33	0.63
2	1.35		3.23		−1.50	1.28	0.17	0.60
3	0.83		4.37			1.65	0.14	0.52
Average model^c^								0.64
Parameter value	0.95	1.13	2.84	−0.04	−0.50			
SE	0.46	1.20	1.64	0.49	0.88			
Variable weight		0.60	0.86	0.17	0.37			
								
Diversity								
Competing model								
1	0.52		0.94			0.00	0.23	0.30
2	0.82				−0.70	0.25	0.20	0.29
3	0.68		0.62		−0.40	0.68	0.16	0.38
Average model								0.38
Parameter value	0.68	0.03	0.45	−0.06	−0.30			
SE	0.16	0.18	0.51	0.02	0.36			
Variable weight		0.19	0.56	0.23	0.54			
								
Composition								
Competing model								
1	−0.20	−1.46		1.64		0.00	0.25	0.62
2	−0.82			1.63	1.34	0.61	0.18	0.61
3	−0.43	−1.03		1.38	0.85	1.94	0.094	0.65
4	−0.69			2.36		1.97	0.093	0.51
Average model								0.66
Parameter value	−0.33	−0.76	−0.42	1.26	0.62			
SE	0.47	0.90	0.94	0.93	0.83			
Variable weight		0.54	0.29	0.76	0.48			

Summary of model selection statistics for the competing models and model-averaged coefficients predicting bee abundance, diversity, and community composition as a function of land cover variables measured in the 1500 m surrounding study sites.

a Wind represents the variable wind pollinated annual crops.

b FA represents flowering annual crops potentially visited by bees.

c For the averaged model the R^2^ is a pseudo-R^2^ derived from regressing the observed values versus model-averaged predictions.

The best model for bee diversity included only one variable, the proportion of forest in the landscape (Table 1). Again, multiple competing models were present in the model set for pollinator diversity and model-averaged coefficients calculated with shrinkage were used to estimate bee diversity in GIS (Table 1, Fig 3B). Model-averaged coefficients for bee diversity were also generally positive for grassland and forest and negative for wind-pollinated crops and flowering annual crops.

Bee community composition, as reflected by the first axis in the NMDS ordination, was best explained by the proportion of grassland and wind-pollinated crops in the surrounding landscape (Table 1). Given the large set of competing models, model-averaged coefficients were used to predict community composition in GIS (Table 1, Fig. 3C). Generally, grassland and forest had negative model-averaged coefficients for this factor, while the model-averaged coefficients for wind-pollinated and flowering annual crops were positive. These results agree with results from vector fitting, which are discussed above in the first paragraph of the results, and have an analogous interpretation.

### Projected landscape-level effects of biofuel scenarios on bees

**Figure 4:**
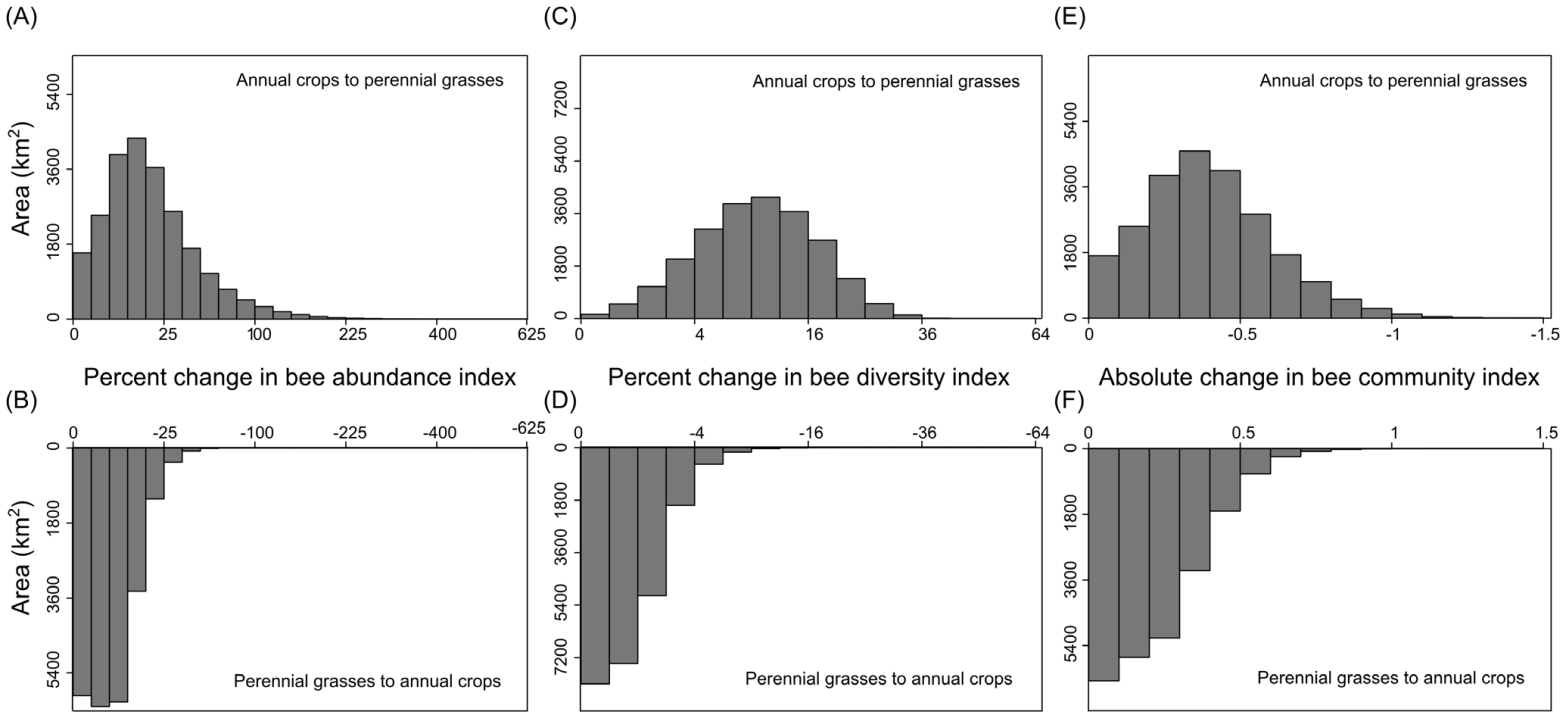
Distribution of percent change for measured bee metrics. The distribution of percent change values calculated under the perennial grassland bioenergy scenario for bee abundance (A), diversity (C), and community composition (E) and under the annual bioenergy scenario for bee abundance (B), diversity (D), and community composition (F). In the annual bioenergy scenario 600,000 ha of marginal grassland was converted into annual bioenergy crops, whereas in the perennial bioenergy scenario 600,000 ha of marginal agricultural land were converted into grassland. Predicted changes in bee communities are only shown for the lower portion of the state where empirical data were collected.

**Figure 5:**
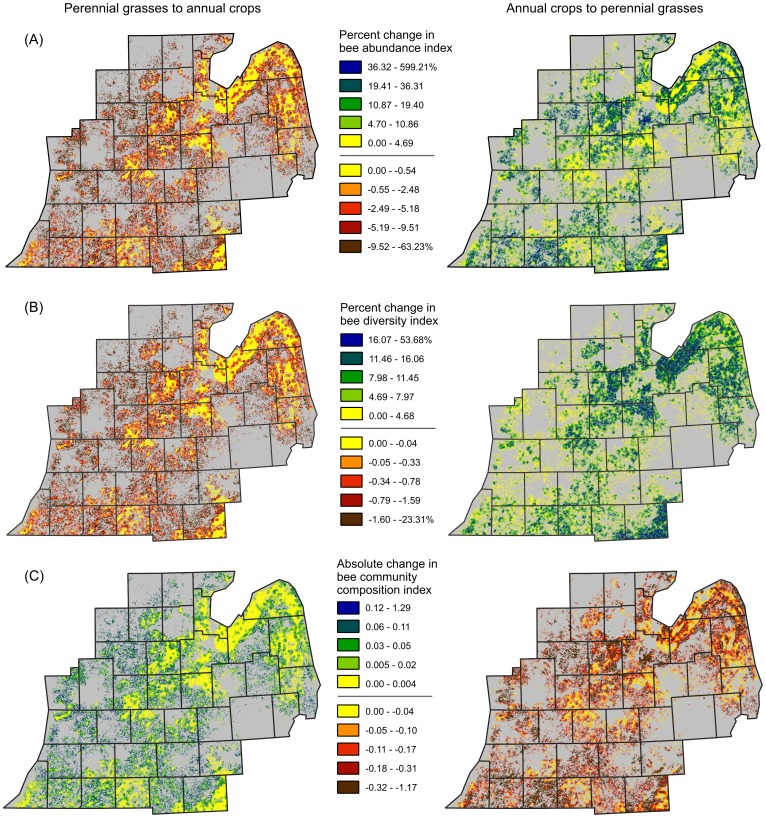
Projected bee metrics. Percent change in bee abundance (A), percent change in bee diversity (B), and difference in community composition (C) predicted for Michigan by an empirical model under annual (left maps) and perennial (right maps) bioenergy production scenarios.

Bee abundance responded positively to the perennial bioenergy scenario and negatively to the annual bioenergy scenario. Under a perennial bioenergy scenario, abundance increased from 0 to 600%, with a landscape-level mean increase of 24% (Fig. 4a). The highest predicted increases in abundance occurred where marginal annual cropland was converted into perennial bioenergy crops (Fig. 5a). Under the annual bioenergy scenario, bee abundance was predicted to decline from 0 to 71%, with a landscape-level mean decrease of 6% (Fig. 4b). The largest loss of abundance is projected to occur in central and western Michigan (Fig. 5a). Areas expected to experience little or no change in abundance, such as pockets of central Michigan and the northwest peninsula of Michigan (Fig. 5A), are regions dominated by prime agricultural soils that are not available for conversion into either perennial or annual bioenergy crops. In contrast, bee abundance is expected to change substantially in regions of Michigan where landscapes support grassland and annual crops on marginal lands. For example, in west Michigan wild bee abundance was projected to experience large declines under the annual scenario, whereas increases were predicted for north-central and southwest Michigan under the perennial scenario.

Similar to the results found for abundance, bee diversity was predicted to increase under the perennial bioenergy scenario and decrease under the annual bioenergy scenario. Bee diversity was predicted to increase from 0 to 53% (Fig. 4c), with a landscape-level mean increase of 10%, under the perennial bioenergy scenario (Fig. 5b). In contrast, bee diversity was predicted to decrease from 0 to 28% (Fig. 4d), with a landscape-level mean decrease in diversity of only approximately 1% under the annual bioenergy scenario (Fig. 5b). Increases in bee diversity under the perennial scenario were predicted for the counties in the peninsula neighboring Lake Michigan, while declines in bee diversity were not predicted for this area of Michigan under the annual scenario. This region of Michigan is an area of intensive agriculture, meaning that areas of annual crops on marginal soils were available for conversion into perennial bioenergy crops, with potential for increasing pollinator diversity. However, areas of marginal grassland were lacking in this region, preventing land conversion into annual bioenergy crops.

Community composition is also predicted to respond to contrasting bioenergy production scenarios. Change in community composition under the perennial scenario, based on NMDS axis one scores, ranged from 0 to −1.73 under the perennial bioenergy scenario (increasing bee abundance and diversity, Fig. 4E), while changes in scores ranged from 0 to 1.37 (decreasing bee abundance and diversity, Fig. 4F) under the annual scenario. Under the perennial scenario, shifts in composition were predicted to occur in north central, western, and south central Michigan (Fig. 5c). Bee composition scores under the annual scenario were expected to change predominantly in west Michigan (Fig. 5c) where bee communities are expected to experience species declines.

## Discussion

Biofuel policies set at the national level are expected to expand biofuel crop production [5], [43], causing substantial changes in land cover across the Midwest [44], [45]. Policies have the potential to influence crop choice as well as crop placement within the landscape, shaping land cover change in ways that are predicted to affect biodiversity and the provisioning of ecosystem services. Our study shows that bee abundance, diversity, and community composition are sensitive to changes in land cover. We found that bee abundance and diversity were greater where there was a greater proportion of grassland and forest in the landscape and were lower where annual agriculture was more prevalent. These results were used to make predictions about how bees in agricultural landscapes would change as annual or perennial bioenergy crops expanded across the region. In a scenario where perennial grassland bioenergy crops are favored, we expect bee abundance and diversity to increase, with shifts to communities that are more dominated by wild bees. In contrast, if policies and markets favor increased adoption of annual bioenergy crops, we predict a reduction in bee abundance and diversity, with community composition moving towards fewer species dominated by generalists such as *A. mellifera*.

In the results reported here, the bee-related metrics were sensitive to landscape-scale land cover change. Mean values reported for increases and decreases in bee abundance and diversity were calculated using all grassland and agricultural pixels across our study region, whether or not they were changed under each scenario. As a result, the magnitudes of the reported percent changes (landscape-level) are smaller than they would be if they were calculated only for the pixels adjacent to those selected for land use change (local-level). At a local level, mean bee abundance increased by 40% under the perennial crop scenario and decreased by 14% under the annual crop scenario. Interestingly, local mean values for bee diversity remained relatively unchanged, with a 9% increase (landscape-scale increase 10%) under the perennial crop scenario and a 2.6% decrease (landscape-scale decrease 1%) under the annual crop scenario. These results suggest that the local effects of bioenergy crop production are more pronounced for bee abundance than for bee diversity.

Forested land played an important role in explaining bee abundance and bee diversity, having the highest variable weight in both sets of models (Table 1). Grassland, however, had the second highest variable weight when explaining bee abundance but the lowest variable weight for bee diversity. The significant relationship between grassland cover and bee abundance may explain why strong local effects are predicted for bee abundance under the perennial and annual land change scenarios but not for bee diversity. The strongest driver of bee diversity was forest cover, and the proportion of forest in the landscape was not changed under either bioenergy scenario. This might explain why switching energy crops had limited local effects on bee diversity. Although our results suggest that increasing perennial grassland bioenergy production will have local effects on bee abundance, conserving forest habitat will be important for maintaining landscape-level bee diversity.

The response of the bee community to annual and perennial bioenergy scenarios was similar for bee abundance and diversity. In general, the bee community under annual bioenergy production shifted to a community composed of fewer bee species, while the bee community under perennial grassland production transitioned to a more diverse community of wild bees (Text S2). The effect of annual bioenergy crop production on bee community composition is of particular interest in the western counties of Michigan where there is significant production of pollinator-dependent fruit and vegetable crops. Because diverse pollinator communities are expected to provide more reliable pollination services by containing redundant [46] or complimentary [47] pollinator species, a shift to annual biofuel production may lead to a decline in bee diversity, with potential effects on the pollination services provided to fruit growers in this region. The relative location of land used for pollinator-dependent crops and biofuels would be expected to influence the degree to which these changes might affect crop yield.

Changes in bee abundance, diversity, and community composition under the perennial bioenergy scenario highlight where opportunities and challenges for grassland bioenergy production exist across Michigan. Several counties located in north-central and south-east Michigan show little or no change in bee abundance, diversity, or community composition. The lack of change in these counties is due to the presence of fruits, vegetables, corn, and soybeans on prime agricultural soils, yielding few opportunities for perennial grassland bioenergy production on marginal lands. In some cases, small isolated patches of perennial habitats may actually serve as population sinks for bees [48], [49], suggesting that one challenge for perennial bioenergy production will be to determine how the size and position of these crops within the landscape will influence pollination services. While placement of bioenergy plantings across the landscape might present challenges, an opportunity exists to target the placement of perennial bioenergy crops near pollinator dependent crops in an effort to increase pollinators and potentially augment pollination services and crop yield.

While the models developed here give insights into the possible effects of future bioenergy production on bees, the resulting maps and our interpretation of these maps depend on several assumptions. First, the models used to predict bee abundance, diversity, and community composition were based on empirical data collected from the lower portion of Michigan and may not extend to other parts of the Midwest. Furthermore, visitation to sunflower measured during the summer may not serve as a good proxy for other pollinator dependent crops, especially early season crops. However, many of the bees we collected, including those in the genus *Bombus* and *Halictus*, are generalists that are present throughout the growing season. Second, conclusions regarding the effect of perennial and annual bioenergy production assume that management practices currently employed for annual crops and perennial grassland habitats do not change substantially with increased bioenergy production. Increasing insecticide use to control emerging pests or annual harvest of bioenergy grasslands could affect bees in ways not reflected in our models. Third, forest was an important variable explaining bee abundance, diversity, and community composition in our study. Under the contrasting bioenergy scenarios developed here, we assumed the proportion of forest remains constant across the landscape, suggesting future loss of forest habitat due to agricultural intensification or urbanization could alter model predictions. Finally, while increasing bee abundance and diversity are generally correlated with higher rates of pollination [28], [50], [51], we recognize that transitioning the landscape into perennial grassland bioenergy production may not translate into increased pollination services, especially if one effective pollinator species is capable of persisting under both scenarios. However, biologically diverse pollinator communities play an important role in providing stable pollination services [52], [53], [54], suggesting that land conversion to perennial grassland bioenergy crops can contribute to supporting services in regions where bees have experienced declines.

## Conclusions

Using field observations, we generated empirical models and predicted bee abundance, diversity, and community composition across Lower Michigan for two contrasting bioenergy production scenarios. From these analyses, we identified areas where bees are expected to benefit substantially from bioenergy policies that promote perennial grassland production, as well as areas where further land conversion to annual bioenergy crops is likely to produce significant challenges for the persistence of diverse bee communities. The methods and models developed here have application for the identification of area thresholds required to maximize biodiversity conservation and target areas of the landscape where perennial bioenergy plantings could facilitate pollination services. However, given market values for annual commodity crops, conversion to perennial grassland bioenergy production will likely be limited without policy changes [55]. Policies that acknowledge the value of biodiversity and the services it provides will be necessary for implementing bioenergy production systems that balance trade-offs between crop production and the support of ecosystem services [56].

## Supporting Information

Text S1 **Results from the test of spatial autocorrelation.**
(DOCX)

Text S2 **Results from the SIMPER analysis including the NMDS ordination with species plotted and a table listing the contribution each species makes to differences in community composition.**
(DOCX)
