# Supplementary material for: Modeling Pollinator Community Response to Contrasting Bioenergy Scenarios
Source: PLoS One. 2014 Nov 3;9(11):e110676. doi: 10.1371/journal.pone.0110676 (PMC4217732; doi:10.1371/journal.pone.0110676)
Supplement: Text S1 — Results from the test of spatial autocorrelation. (DOCX) [file pone.0110676.s001.docx]

**Supporting Information: Text S1**

Figure S1. Moran’s I was used to test for spatial autocorrelation between study sites using residuals from the AICc-determined best model for bee abundance, diversity, and community composition. No evidence of spatial autocorrelation was detected for bee abundance (A), diversity (B), or community composition (C).
