# Supplementary material for: Modeling Pollinator Community Response to Contrasting Bioenergy Scenarios
Source: PLoS One. 2014 Nov 3;9(11):e110676. doi: 10.1371/journal.pone.0110676 (PMC4217732; doi:10.1371/journal.pone.0110676)
Supplement: Text S2 — Results from the SIMPER analysis including the NMDS ordination with species plotted and a table listing the contribution each species makes to differences in community composition. (DOCX) [file pone.0110676.s002.docx]

**Supporting Information: Text S2**

Figure S2. Bee species are plotted in ordination space to highlight which species are correlated with sites surrounded by high proportion of grassland and forest compared to sites surrounded by high proportions of annual agriculture. Species labels indicate the most abundance species and less abundant species are not included when labels overlap.


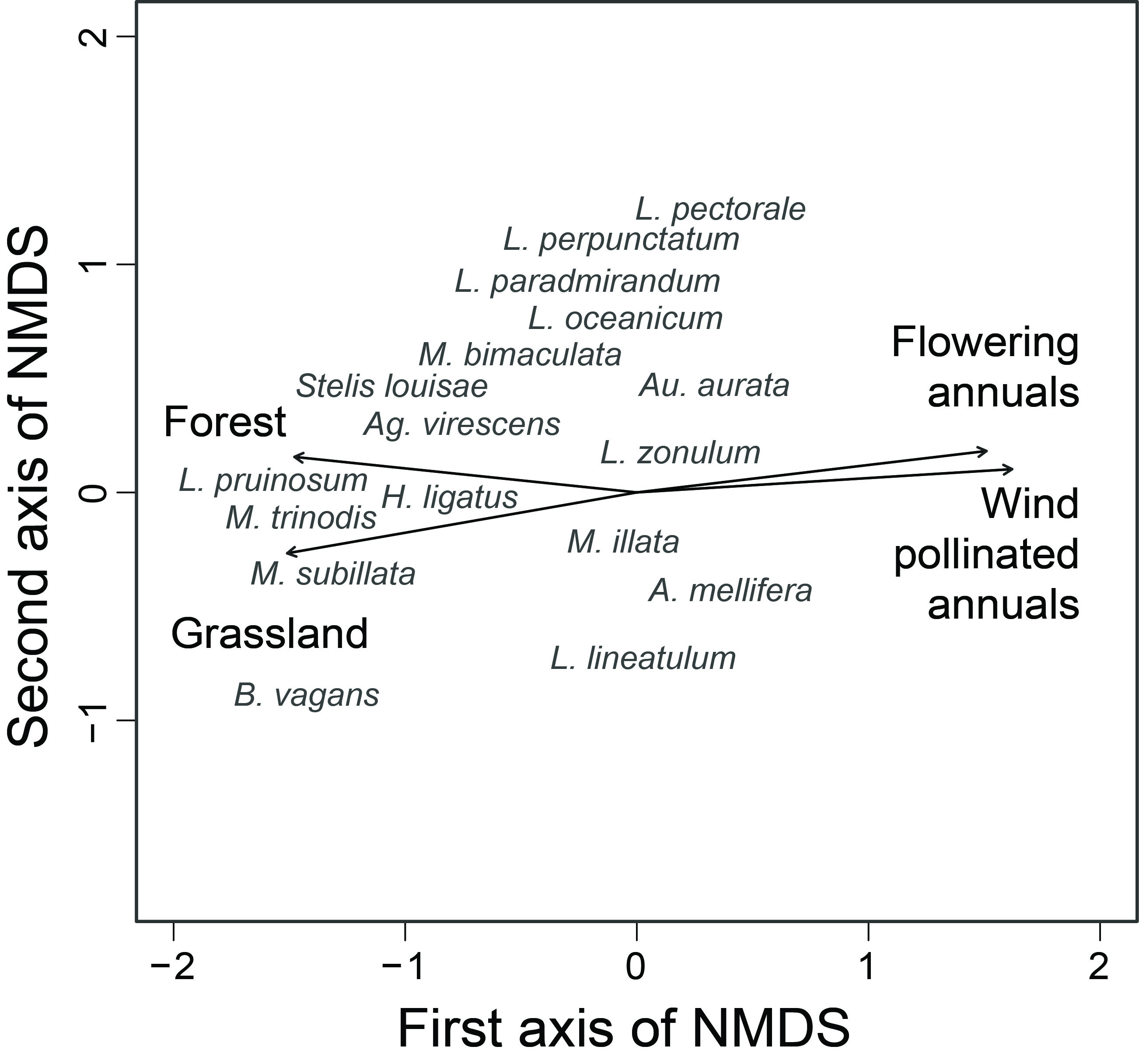


Table S2. The simper analysis identifies differences in community among sites and compared sites with NMDS axis one scores greater than zero to those with NMDS scores less than zero. Bee species are rank ordered with the highest contrast values contributing the most to differences between sites with positive (i.e. increasing annual agriculture) and negative (i.e. increasing grassland and forest) NMDS scores.

| Species | Contribution | SD |
| --- | --- | --- |
| *Apis mellifera* Linnaeus | 0.17 | 0.14 |
| *Augochlorella aurata* (Smith) | 0.08 | 0.1 |
| *Halictus ligatus* Say | 0.07 | 0.1 |
| *Bombus impatiens* Cresson | 0.05 | 0.05 |
| *Lasioglossum zonulum* (Smith) | 0.05 | 0.08 |
| *Lasioglossum paradmirandum* (Knerer & Atwood) | 0.04 | 0.1 |
| *Agapostemon virescens* (Fabricius) | 0.04 | 0.08 |
| *Lasioglossum pilosum* (Smith) | 0.03 | 0.03 |
| *Melissodes subillata* LaBerge | 0.02 | 0.04 |
| *Melissodes agilis* (Cresson) | 0.02 | 0.04 |
| *Lasioglossum versatum* (Robertson) | 0.02 | 0.03 |
| *Melissodes illata* Lovell & Cockerell | 0.02 | 0.04 |
| *Melissodes bimaculata* (Lepeletier) | 0.02 | 0.07 |
| *Bombus vagans* Smith | 0.02 | 0.04 |
| *Andrena helianthi* Robertson | 0.01 | 0.04 |
| *Lasioglossum pruninosum* (Robertson) | 0.01 | 0.04 |
| *Lasioglossum sagax* (Sandhouse) | 0.01 | 0.04 |
| *Lasioglossum leucocomum* (leucocomum) | 0.01 | 0.02 |
| *Lasioglossum oceanicum* (Cockerell) | 0.01 | 0.02 |
| *Heriades leavitti* Crawford | 0.01 | 0.04 |
| *Lasioglossum perpuncatum* (Ellis) | 0.01 | 0.02 |
| *Xylocopa virginica (Linnaeus)* | 0.009 | 0.02 |
| *Hylaeus affinis/modestus* (Smith) | 0.009 | 0.03 |
| *Melissodes trinodis* Robertson | 0.009 | 0.03 |
| *Lasioglossum lineatulum* (Crawford) | 0.008 | 0.03 |
| *Anthidium manicatum* (Linnaeus) | 0.008 | 0.03 |
| *Ceratina mikmaqi* Rehan & Sheffield | 0.008 | 0.03 |
| *Halictus rubicundus* (Christ) | 0.008 | 0.02 |
| *Lasioglossum pectorale* (Smith) | 0.008 | 0.02 |
| *Lasioglossum bruneri* (Crawford) | 0.008 | 0.02 |
| *Megachile pugnata* (Say) | 0.006 | 0.02 |
| *Stelis louisae* Cockerell | 0.006 | 0.02 |
| *Megachile latimanus* Say | 0.003 | 0.01 |
| *Agochloropsis metallica* (Fabricius) | 0.003 | 0.01 |
| *Lasioglossum imitatum* (Smith) | 0.003 | 0.01 |
| *Lasioglossum zephyrum* (Smith) | 0.003 | 0.01 |
| *Lasioglossum leucozonium* (Schrank) | 0.003 | 0.01 |
| *Lasioglossum laevissimum* (Smith) | 0.003 | 0.01 |
